# Intrauterine Device Use: A New Frontier for Behavioral Neuroendocrinology

**DOI:** 10.3389/fendo.2022.853714

**Published:** 2022-07-22

**Authors:** Adriene M. Beltz, Michael I. Demidenko, Natasha Chaku, Kelly L. Klump, Jane E. Joseph

**Affiliations:** ^1^ Department of Psychology, University of Michigan, Ann Arbor, MI, United States; ^2^ Department of Psychology, Michigan State University, East Lansing, MI, United States; ^3^ Department of Neurosciences, Medical University of South Carolina, Charleston, SC, United States

**Keywords:** brain function, connectivity, fMRI, gender, intrauterine device, networks, oral contraceptive, spatial skills

## Abstract

Intrauterine devices (IUDs) are the most-used reversible contraceptive method for women in the world, but little is known about their potential modulation of brain function, cognition, and behavior. This is disconcerting because research on other hormonal contraceptives, especially oral contraceptives (OCs), increasingly shows that exogenous sex hormones have behavioral neuroendocrine consequences, especially for gendered cognition, including spatial skills. Effects are small and nuanced, however, partially reflecting heterogeneity. The goal of this paper is to introduce IUD use as a new frontier for basic and applied research, and to offer key considerations for studying it, emphasizing the importance of multimodal investigations and person-specific analyses. The feasibility and utility of studying IUD users is illustrated by: scanning women who completed a functional magnetic resonance imaging mental rotations task; taking an individualized approach to mapping functional connectivity during the task using network analyses containing connections common across participants and unique to individual women, focusing on brain regions in putative mental rotations and default mode networks; and linking metrics of brain connectivity from the individualized networks to both mental rotations task performance and circulating hormone levels. IUD users provide a promising natural experiment for the interplay between exogenous and endogenous sex hormones, and they are likely qualitatively different from OC users with whom they are often grouped in hormonal contraceptive research. This paper underscores how future research on IUD users can advance basic neuroendocrinological knowledge and women’s health.

## Introduction

There has been a recent uptick in the biopsychological study of hormonal contraceptives, partially reflecting women’s increased scientific participation and funding emphases (1–4). Indeed, hormonal contraceptives are not only important for their contraceptive and medical benefits, but also as a natural experiment for exogenous sex hormone influences on the brain, cognition, and behavior, which are severely under-studied domains of women’s health. Hormonal contraceptives come in many forms, with intrauterine devices (IUDs) being the most-used worldwide (159 million users; 5). Oral contraceptives (OCs), however, are the most widely-studied form, likely owing to their prevalence in North America and Europe (5). Thus, there are perplexing knowledge gaps regarding neuroendocrine links to cognition and behavior in IUD users. This paper presents vital considerations for filling these gaps and illustratively showcases how multimodal study designs and person-specific methods have potential to accurately reflect the heterogeneity present–but often erroneously ignored–among all women, particularly in relation to ovarian hormone influences (e.g., 6).

Most empirical research on hormonal contraceptives considers users to be homogenous, and thus, combines women using different forms (e.g., IUDs, OCs, and implants; 2–4, 7). This is problematic because hormonal contraceptives have varying exogeneous hormone constituents and doses, and thus, have varying influences on endogenous hormone levels. For instance, combined OCs contain a synthetic estrogen (usually ethinyl estradiol) and a progestin varying in androgenicity, from anti-androgenic to highly androgenic. In many monophasic formulations, women receive stable doses of both hormones for 21 days followed by a placebo for 7 days (although schedules vary). In many triphasic formulations, women receive consistent doses of ethinyl estradiol for 21 days with progestin doses increasing slightly every 7 days for 3 weeks, followed by a placebo for 7 days. The pills alter endogenous ovarian hormone secretion through negative feedback mechanisms and prevent pregnancy by inhibiting ovulation. Most IUDs, however, release a relatively constant dose of the progestin levonorgestrel, which is moderately-to-highly androgenic, for up to three or five years (8). They prevent pregnancy by instigating local changes to reproductive biology (e.g., in tissue within the endometrial cavity), and their systemic impacts on endogenous ovarian hormone levels (especially because they do not contain estradiol), and on brain function and behavior, are unclear. Their reported side effects, however, include acne, headaches, and breast tenderness (8), and women using IUDs have shown risks for depression similar to OC users (9), suggesting that effects may be systemic.

Thus, there is significant heterogeneity among hormonal contraceptives. This heterogeneity is exacerbated by the established heterogeneity in women’s neuroendocrine function, including in receptor sensitivity and in lifestyle factors that affect hormone function (10, 11). It is, therefore, not surprising that research on the neural, cognitive, and behavioral consequences of hormonal contraceptive use offers only a few consistent results. One of them concerns depression, as noted above (9). Another concerns OCs and spatial skills. OC progestin androgenicity has been positively associated with three-dimensional (3D) mental rotations performance (12–15), which shows a large gender difference in which men–on average–outperform women (see 1). There is also indication that OC ethinyl estradiol dose is inversely related to mental rotations performance (12). These findings broadly align with reviews and recent empirical work suggesting that high androgens (and perhaps progestogens) as well as low estradiol may facilitate mental rotations performance in women (1, 2, 4, 16, 17). There are, surprisingly and unfortunately, no studies that focus on mental rotations performance (or any aspect of cognition) in IUD users as a homogenous group; when they are studied, IUD users are combined with other hormonal contraceptive users, increasing heterogeneity and limiting inferences (e.g., 13, 18).

The neural substrates underlying hormonal contraceptives and mental rotations performance are also not well-understood (see 2). Generally, functional magnetic resonance imaging (fMRI) studies show that mental rotation tasks engage occipital and parietal regions and some temporal and frontal regions, especially in the right hemisphere, and these regions are linked to gender differences in task performance (1, 19–21). Men typically recruit visual and parietal regions more strongly than women, and women tend to engage frontal regions, such as the inferior frontal gyrus, more than men. These differences are thought to be related to gender differences in strategy use (22, 23). They may also be linked to testosterone and progesterone, but especially to estrogen, as the hormones have been shown to modulate brain activity underlying spatial task performance across the natural menstrual cycle (24–27).

It is necessary to emphasize, however, that this extant literature overwhelmingly relies on traditional neuroscience methods; studies come from a functional localization perspective and focus on task-related brain regions identified through cognitive subtraction (28). For instance, focus might be on parietal activation during mental rotations versus passive viewing, determined by averaging brain activity across trials and participants, often regardless of their hormone milieus. Although they have led to important findings, these methods can also result in null or inaccurate findings because the brain operates as a network (e.g., different parietal regions communicate with several different frontal regions during rotation; 29), hormone milieus vary within and between individuals (7, 8), and people are heterogeneous in their cognition and behavior (30). A person-specific neural network perspective could overcome these limitations. For instance, although the default mode network, which includes midline and lateral parietal regions as well as the medial prefrontal cortex (31), is more active during rest than tasks, it contributes to cognitive function and task performance (32, 33). Women also appear to have greater connectivity (i.e., synchrony) of default mode regions during rest than do men (32, 34). Interestingly, no work has examined the interplay between the default mode network and a set of regions constituting a putative mental rotations network, especially in relation to sex hormones.

## Feasibility and Utility of Studying IUD Use

There is a pressing need for future research to examine the neuroendocrine underpinnings of links to behavior and cognition, such as mental rotations performance, in IUD users. In doing so, it is vital to conduct multimodal investigations that assess links among hormone levels (e.g., circulating, inferred from hormonal contraceptive dosing, or otherwise marked by hormone activity levels), brain function, and behavior (e.g., mental health reports or cognitive task performance), and to consider heterogeneity among women in those links.

### Multimodal Data Collection

To illustrate the feasibility and utility of a multimodal person-specific approach, data from 11 IUD users is briefly presented (*M_age_* =28.37, *SD_age_* =5.40; 55% White, 27% Asian, 18% Black; 73% non-Hispanic). Participants are from an ongoing fMRI study that was conducted with approval from the University of Michigan Institutional Review Board; all participants provided informed consent. All participants were using slow-release IUDs containing the androgenic progestin levonorgestrel (nine were using Mirena^®^, one Kyleena^®^, and one Skyla^®^). They had been using the IUDs for at least the past three months and had no reproductive health issues (e.g., polycystic ovary syndrome) or previous pregnancies. They were also not using medications containing sex hormones.

Among other study procedures, participants completed a 60-minute online monitored survey and received a 60-minute MRI scan. The morning of the scan, they provided approximately 2mL of saliva, which was collected *via* passive drool within 30 minutes of waking. Saliva samples were assayed using high sensitivity estradiol, progesterone, and testosterone enzyme-linked immunosorbent assay kits according to manufacturer instructions (35) by the Core Assay Facility at the University of Michigan. They were assayed in duplicate and averaged for analyses. See **Supplementary Materials** for details, including assay sensitivities and intra-assay coefficients of variation. The top third of **Table 1** shows means and standard deviations (in pg/mL) for all three hormones. These hormone levels do not appear to be suppressed, as are hormone levels in OC users (e.g., 36); in fact, progesterone in IUD users may be elevated compared to both naturally cycling women and OC users (e.g., 37). Thus, these data are consistent with insinuations that IUDs have systemic effects.

**Table 1:** Multimodal data for IUD users (n=11): Endogenous hormone levels, mental rotations task performance, and person-specific network densities.

Hormone Assessments (in pg/mL)	IUD Users	
	*M*	*SD*
Estradiol	1.33	.71
Progesterone	239.01	124.99
Testosterone	132.82	64.88
**In-Scanner Behavior**		
Mental Rotations Performance (% correct)	75.00	8.39
**Neural Network Densities**		
Total network complexity	35.45	4.53
Within-MRN density (proportion of total)	.34	.03
Within DMN density (proportion of total)	.18	.03
Between-network density (proportion of total)	.19	.05

IUD, intrauterine device; M, mean; SD, standard deviation.

During each scan, participants completed two unique runs of a slow event mental rotations task (27). Each run contained 16 trials during which participants determined whether a pair of 2D or 3D objects formed from small blocks were accurate rotations of each other. The 3D condition was based on the traditional Shepard and Metzler task (38), and the 2D condition controlled for basic visual processing, decision-making, and rotation. Task timing is shown in **Supplemental Figure S1**. Each run lasted 4 min 24s, and correct responses were recorded. Behavior is vital to the interpretation of brain function, and the middle third of **Table 1** shows that IUD users correctly identified whether the rotated 2D or 3D objects were the same in 75% of trials, on average.

Neuroimaging data were acquired using a GE Discovery MR750 3.0 Tesla scanner with a standard coil (Milwaukee, WI). Structural data consisted of 208 slices from a T1 SPGR PROMO sequence (TI=1060ms, TE=Min Full, flip angle=8°, FOV=25.6 cm, slice thickness=1mm, 256x256 matrix, interleaved). Before the task, a fieldmap was acquired using a spin-echo EPI sequence (TR=7400ms, TE=80ms, FOV=22.0cm, 64x64 matrix, interleaved). Functional data consisted of 40 interleaved slices collected during an EPI sequence (TR=2000ms, TE=25 ms, flip angle=90°, FOV=22.0 cm, slice thickness=3mm, 64x64 matrix, 134 volumes). Standard preprocessing was conducted, as described in **Supplementary Materials**. Blood oxygen level-dependent (BOLD) time series were then extracted from ten regions of interest (ROIs) with 10mm diameters, four that constituted the default mode network (DMN) and six that constituted a putative mental rotations network (MRN; see **Supplemental Table S1** for central coordinates), following past work (2, 27, 39). Individual differences in anatomical structure were addressed by intersecting ROIs with participants’ binarized grey matter masks (generated using FSL’s FAST; 40). Time series from the two runs were concatenated after processing.

### Person-Specific Functional Connectivity

Person-specific connectivity analyses were conducted on the mental rotations task-related fMRI data in order to reveal potential individual differences in the neuroendocrinology of IUD use. Specifically, the BOLD time series for each participant was submitted to group iterative multiple model estimation (GIMME), which has been validated in extensive largescale simulations (e.g., 41). Details can be found in tutorials (42, 43) and empirical applications (e.g., 6, 44, 45). Briefly, GIMME uses a data-driven approach based on Lagrange Multiplier tests to add directed contemporaneous (same-volume) or lagged (from one volume to the next) connections to participants’ null networks (with no connections). In this application, GIMME added group-level connections (reflecting systematic effects of IUDs) that were significant for at least 75% of the sample to the networks of all women in the sample, followed by individual-level connections (reflecting heterogeneity) for each woman until the model fit well according to standard indices. All connections (i.e., whether at the group- or individual-level) were fit uniquely to each woman’s data, and thus, have individualized weights. Each participant’s network was then characterized by its overall complexity (i.e., number of connections) as well as its subnetwork densities (i.e., number of network connections divided by complexity): within the MRN, within the DMN, and between the MRN and DMN.

**Figure 1:**
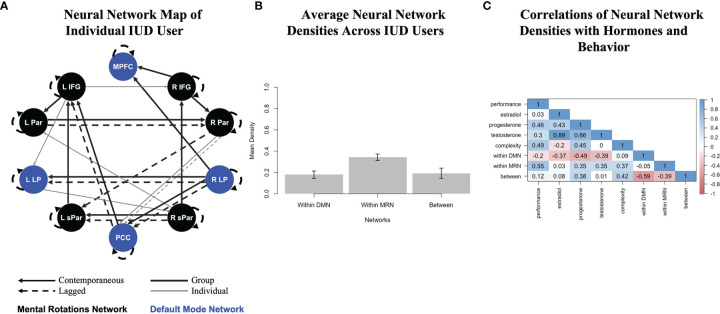
Analysis pipeline linking multimodal data in IUD users who completed a mental rotations fMRI task and provided saliva for hormone assays. **(A)** A person-specific neural network generated by group iterative multiple model estimation (GIMME) for one IUD user. Black nodes show putative mental rotations network regions, and blue nodes show default mode network regions. Solid lines are contemporaneous (same-volume) connections, and dashed lines are lagged (next-volume) connections. Thick black lines are group-level connections significant for at least 75% of the sample, but estimated for all IUD users, and thin gray lines are individual-level connections unique to this IUD user; all participants had corresponding estimated networks (though not depicted here). All connections also have a direction (positive or negative) and beta weight associated with them (also not depicted here). This woman’s network fit her functional data well (*χ^2^* (112)=652.60, *p*<.001, RMSEA=.135, SRMR=.039, CFI=.955, NNFI=.924). R, right; L, left; IFG, inferior frontal gyrus; Par, parietal; LP, lateral parietal; sPar, superior parietal; MPFC, medial prefrontal cortex; PCC, posterior cingulate cortex. **(B)** Average neural network densities extracted from the person-specific GIMME networks of all IUD users (and divided by overall network complexity), with error bars showing standard deviations. **(C)** Correlations among multimodal data, including mental rotations task performance in the scanner, endogenous hormone levels, and neural network features, including overall complexity and network densities. Color-coded correlations are shown in the matrix, with dark red reflecting strong inverse relations through dark blue reflecting strong positive relations.

The 11 person-specific neural networks generated by GIMME fit the data well, as indicated by average fit indices: *χ^2^* (109.55)=554.17, *p*<.001, RMSEA=.121, SRMR=.036, CFI=.957, NNFI=.926. **Figure 1A** presents the network for one individual IUD user. Black nodes represent MRN ROIs, and blue nodes represent DMN ROIs. The network reflects homogeneity, as it prioritized contemporaneous (solid lines) and lagged (dashed lines) group-level connections consistent across all IUD users, which are shown as thick black lines. Notice that most group-level connections are between contralateral ROIs (e.g., left and right parietal, lateral parietal, and superior parietal) or ROIs in the same network; only a few are between ROIs in different networks (e.g., from the posterior cingulate cortex to the left inferior frontal gyrus). Heterogeneity was also reflected in the contemporaneous and lagged individual-level connections unique to this participant, which are shown as thin gray lines in **Figure 1A**. For this woman, complexity was 33, and the MRN and DMN densities were 33 and 15, respectively, with a 21 between-network density. The bottom third of **Table 1** shows average complexity and network densities across all IUD users, and **Figure 1B** also graphically shows the average densities. As expected for these task-related fMRI data, the density of connections within the MRN was greater than within the DMN or between the two networks.

Finally, **Figure 1C** shows how neural network densities were related to multimodal study data, including in-scanner mental rotations task performance and endogenous hormone levels. Task performance was positively related to overall neural network complexity, especially to the density of the MRN, as well as to progesterone and even testosterone levels, which were correlated with each other, consistent with the androgenic pharmacokinetic properties of the progestin-based IUDs being used by this sample. The density of the DMN, however, was inversely correlated with all hormones.

## Discussion

IUD users provide a novel and promising natural experiment for neuroendocrinological research and are prevalent worldwide (5), but they remain understudied. Research on IUD users–as an independent group not combined with other hormonal contraceptive users–is necessary and feasible. It is necessary because IUDs have functional properties that inherently differ from those of other hormonal contraceptives, such as OCs, which suppress endogenous hormones levels and inhibit ovulation (36). In fact, the illustrative data presented here indicate that circulating progesterone may be enhanced in IUD users. More work is sorely needed to determine the extent to which salivary assays of endogenous hormones reflect or are modulated by intrauterine administrations of synthetic hormones, and this work must consider different data collection methods (e.g., saliva versus serum) and analysis approaches (e.g., ELISA versus mass spectrometry; 46).

Moreover, research with IUD users is arguably more feasible than research on ovarian hormones *via* menstrual cycle phase comparisons in naturally cycling women or even *via* active versus placebo pill comparisons in OC users, as it does not require repeated assessments or phase monitoring, which is not only difficult, but often inaccurate (7).

When studying the neural consequences of the interplay between exogenous and endogenous hormones in IUD users, behavioral assessments and heterogeneity are vital to consider. Regarding behavior, it is prudent to examine behaviors that have already been linked to hormonal contraceptives outside of the scanner, such as mental rotations performance, in order to reveal underlying neural mechanisms (1, 2, 4). Utilizing tasks that maximize power is also important for detecting robust and reliable effects (47, 48). The mental rotations task used in this feasibility demonstration was statistically powerful because it contained 3D (experimental) and 2D (control) conditions instead of a control condition that did not require rotation (see 27).

Regarding neural heterogeneity, multivariate connectivity analyses that incorporate individual differences (see 48), or better yet, person-specific effects, are well-suited to capturing multimodal associations in IUD users; in this way, GIMME has particular utility (41–43). As seen in the illustrative analysis within this paper, GIMME mapped connections among ROIs in the MRN and DMN in a data-driven way, such that only the most meaningful ROI connections were added to participants’ individualized networks. Specifically, if model parameters indicated that certain connections were statistically informative for most IUD users, then those connections were estimated uniquely in all women’s networks based on their own time series. Thus, GIMME provided group-level inferences without averaging! This has incredible utility for future studies of IUD users–and of other heterogenous samples–as human neuroendocrine processes are unique due to individual differences in biology (e.g., hormone receptor sensitivity; 10), psychology (e.g., emotion; 49), and context (e.g., modulation by stress; 11). Averaging across these heterogeneous samples can falsely exaggerate findings, cancel out effects, or distort inferences (30). Person-specific networks, though time-intensive and complex, are more likely to accurately reflect neuroendocrine nuances.

### Conclusions

The goal of this paper was to highlight the value of IUD users as a natural experiment for studying both exogenous and endogenous sex hormone links to gendered neurocognition (namely, mental rotations), by utilizing multimodal research designs and person-specific approaches to the analysis of fMRI data. Future investigations should focus on IUD users as an independent group; it may rarely be appropriate to combine IUD users with OC users to create a general “hormonal contraceptive” group. Future investigations should also triangulate hormonal, neural, and behavioral data, and analyze these data in ways that accurately reflect heterogeneity within IUD users, who have unique neuroendocrine milieus. Indeed, effects of IUD use are likely to be both systemic within women, and unique to individual women. This means that future investigations are important for both revealing ovarian hormone influences on the brain and behavior, and for advancing multimodal and person-specific methods within behavioral neuroendocrinology.

## Data Availability Statement

The datasets presented in this article are not readily available because data use agreements need to be established. Requests to access the datasets should be directed to Adriene Beltz, abeltz@umich.edu.

## Ethics Statement

The studies involving human participants were reviewed and approved by University of Michigan IRB (Health Sciences and Behavioral Sciences). The patients/participants provided their written informed consent to participate in this study.

## Author Contributions

AB conceptualized and directed the study with critical input from KK and JJ; MD helped collect the data; AB and MD analyzed the data with critical input from NC; AB, MD and NC drafted the manuscript; all authors provided critical revisions and approved the final version.

## Funding

AB was supported by the Jacobs Foundation, MD and NC by National Institutes of Health (NIH) grant T32HD007109 (PIs: C. Monk, V. McLoyd, and S. Gelman), KK by NIH grant R01MH111715, and JJ by NIH grants P20RR015592 (PI: T. Curry) and K12DA014040 (PI: E. Wilson).

## Conflict of Interest

The authors declare that the research was conducted in the absence of any commercial or financial relationships that could be construed as a potential conflict of interest.

## Publisher’s Note

All claims expressed in this article are solely those of the authors and do not necessarily represent those of their affiliated organizations, or those of the publisher, the editors and the reviewers. Any product that may be evaluated in this article, or claim that may be made by its manufacturer, is not guaranteed or endorsed by the publisher.
