# Supplementary material for: Intrauterine Device Use: A New Frontier for Behavioral Neuroendocrinology
Source: Front Endocrinol (Lausanne). 2022 Jul 22;13:853714. doi: 10.3389/fendo.2022.853714 (PMC9352855; doi:10.3389/fendo.2022.853714)
Supplement: Supplementary file 1 [file DataSheet_1.docx]

**Supplemental Materials for**

**“Intrauterine device use: A new frontier for behavioral neuroendocrinology”**

**Hormone Assessments**

To minimize contamination, participants were instructed to refrain from eating, drinking, smoking, chewing gum, or brushing their teeth before providing their sample each morning. Samples were placed in insulated pouches and stored on ice in participant freezers until the MRI session. Upon arrival, samples were stored at -20° C until analysis. Assay sensitivities for the plate were 16.07pg/mL±0.94 (high control range) and 6.37pg/mL±1.18 (low control range) for estradiol; 768.89pg/mL±61.87 (high control range) and 42.67pg/mL±4.94 (low control range) for progesterone, and 224.35pg/mL±9.16 (high control range) and 15.37pg/mL±1.89 (low control range) for testosterone. Intra-assay coefficients of variation were 5.90 for estradiol, 6.74 for progesterone, and 4.77 for testosterone. All raw hormone values were visually inspected for extreme values likely attributable to assay contamination or measurement error.

**fMRI Acquisition, Preprocessing, and ROI Extraction**

Functional data were reconstructed, images were realigned, and field map correction was applied in SPM12; physiological noise was removed using RETROICOR (Glover, 2000). Data were then preprocessed using FSL (www.fmrib.ox.ac.uk/fsl; FMRI Expert Analysis Tool Version 6.00), including (Soares et al., 2016): registration of functional data to high resolution and standard MNI space using FLIRT (Jenkinson, 2001; Jenkinson et al., 2002), motion correction using MCFLIRT (Jenkinson et al., 2002), slice-time correction using Fourier-space time-series phase shifting, non-brain removal, spatial smoothing using a Gaussian kernel of 6mm FWHM, grand-mean intensity normalization of the entire 4D dataset by a single multiplicative factor; and highpass temporal filtering (sigma=50.0s).

**References**

Glover, G. H., Li, T. Q., & Ress, D. . (2000). Image-Based Method for Retrospective Correction of Physiological Motion Effects in fMRI: RETROICOR. *Magnetic Resonance in Medicine: An Official Journal of the International Society for Magnetic Resonance in Medicine, 44*(1), 162-167. <https://doi.org/j.neuroimage.2009.05.012>

Jenkinson, M., & Smith, S. (2001). A global optimisation method for robust affine registration of brain images. *Medical image analysis, 5*(2), 143-156. <https://doi.org/10.1016/s1361-8415(01)00036-6>.

Jenkinson, M., Bannister, P., Brady, M., & Smith, S. (2002). Improved Optimization for the Robust and Accurate Linear Registration and Motion Correction of Brain Images. *Neuroimage, 17*(2), 825-841. <https://doi.org/10.1006/nimg.2002.1132>

Soares, J. M., Magalhaes, R., Moreira, P. S., Sousa, A., Ganz, E., Sampaio, A., Alves, V., Marques, P., & Sousa, N. (2016). A Hitchhiker's Guide to Functional Magnetic Resonance Imaging. *Frontiers in Neuroscience, 10*, 515. <https://doi.org/10.3389/fnins.2016.00515>

Table S1. *Central MNI Coordinates for Subnetwork Regions of Interest (ROIs)*

*Used in Person-specific Connectivity Analyses*.

| **Region of Interest** | **Network** | **MNI**  ***x*** | **MNI**  ***y*** | **MNI**  ***z*** |
| --- | --- | --- | --- | --- |
|  |  |  |  |  |
| Right Inferior Frontal Gyrus (R IFG) | Mental Rotations | 36 | 20 | 22 |
| Left Inferior Frontal Gyrus (L IFG) | Mental Rotations | -36 | 20 | 22 |
| Right Parietal Cortex  (R Par) | Mental Rotations | 25 | -62 | 42 |
| Left Parietal Cortex  (L Par) | Mental Rotations | -25 | -62 | 42 |
| Right Superior Parietal  (R sPar) | Mental Rotations | 52 | -40 | 58 |
| Left Superior Parietal  (L sPar) | Mental Rotations | -52 | -40 | 58 |
|  |  |  |  |  |
| Medial Prefrontal Cortex (MPFC) | Default Mode | 0 | 46 | -4 |
| Right Lateral Parietal Cortex (R LP) | Default Mode | 46 | -62 | 32 |
| Left Lateral Parietal Cortex (L LP) | Default Mode | -40 | -68 | 36 |
| Posterior Parietal Cortex (PPC) | Default Mode | -4 | -50 | 40 |

*Note*. 10mm spheres then drawn around central coordinates, which were intersected with individual-level gray matter maps. MNI: Montreal Neurological Institute.


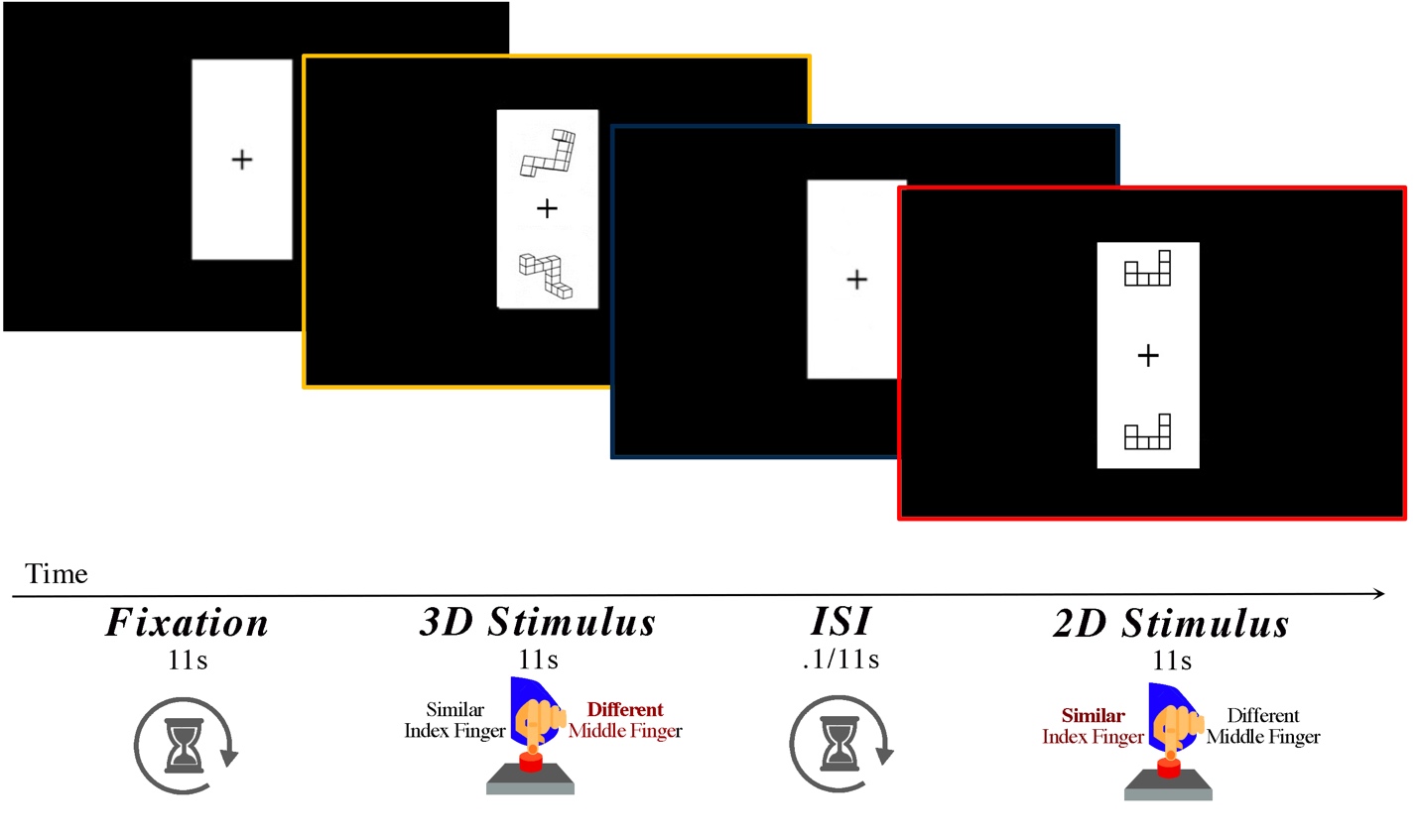


*Figure S1*. Depiction of the mental rotations fMRI task, showing a 11s fixation followed by a 3D trial, 100ms or 11s intertrial interval, and a 2D trial. During each trial, participants indicated whether the two objects were (index button press) or were not (middle finger button press) the same object, potentially rotated in space. 3D and 2D trials were presented in different orders throughout the task. Another fixation cross was presented before the next trial. Each run consisted of 16 trials (8 were 3D trials and 8 were 2D trials), there were two runs per scan session, and two scan sessions per participant. 3D: three-dimensional; 2D: two-dimensional; ISI: intertrial interval.
